# Transcutaneous electrical nerve stimulation for fibromyalgia-like syndrome in patients with Long-COVID: a pilot randomized clinical trial

**DOI:** 10.1038/s41598-024-78651-5

**Published:** 2024-11-08

**Authors:** Alejandro Zulbaran-Rojas, Rasha O. Bara, Myeounggon Lee, Miguel Bargas-Ochoa, Tina Phan, Manuel Pacheco, Areli Flores Camargo, Syed Murtaza Kazmi, Mohammad Dehghan Rouzi, Dipaben Modi, Fidaa Shaib, Bijan Najafi

**Affiliations:** 1 Michael E DeBakey Department of Surgery, Baylor College of Medicine, Houston, TX USA; 2 Center for Advanced Surgical & Interventional Technology (CASIT), Department of Surgery, David Geffen School of Medicine, University of California, Los Angeles, 700 Westwood Plaza, Suite 2200, Los Angeles, CA 90095 USA; 3 Department of Pulmonary Critical Care, Baylor College of Medicine, Houston, TX USA

**Keywords:** Randomized controlled trials, Neuromuscular disease

## Abstract

This study investigated the effect of Transcutaneous Electrical Nerve Stimulation (TENS) for fibromyalgia-like symptoms including chronic widespread musculoskeletal pain, fatigue, and/or gait impairment in twenty-five individuals with long-COVID. Participants were randomized to a high dose (intervention group, IG) or low dose (placebo group, PG) TENS device. Both groups received daily 3–5 h of TENS therapy for 4-weeks. The Brief Pain Inventory assessed functional interference from pain (BPI-I), and pain severity (BPI-S). The global fatigue index (GFI) assessed functional interference from fatigue. Wearable technology measured gait parameters during three 30-feet consecutive walking tasks. At 4-weeks, the IG exhibited a greater decrease in BPI-I compared to the PG (mean difference = 2.61, *p* = 0.008), and improved in gait parameters including stride time (4-8%, test condition dependent), cadence (4-10%, depending on condition), and double-support phase (12% in dual-task) when compared to baseline. A sub-group meeting the 2010 American College of Rheumatology Fibromyalgia diagnostic criteria undergoing high-dose TENS showed GFI improvement at 4-weeks from baseline (mean change = 6.08, *p* = 0.005). Daily TENS therapy showed potential in reducing functional interference from pain, fatigue, and gait alterations in long-COVID individuals. The study’s limited power could affect the confirmation of certain observations. Extending the intervention period may improve treatment effectiveness.

## Introduction

Post-Acute Sequelae of Sars-Cov-2 or long-COVID is a multisystemic condition characterized by persistent symptoms in one or more organs after acute COVID-19 clearance^1^. Approximately 1 in 13 adults recovering from COVID-19 experience long-COVID^2^, with the musculoskeletal system commonly affected^3^. Persistent symptoms include long-lasting widespread pain, fatigue and weakness varying in frequency, duration, and intensity^4,5^, which can lead to functional interference affecting gait and daily activities^6–8^.

The mechanism of musculoskeletal damage in long-COVID remains unknown, and whether widespread pain is associated with the nervous system is uncertain. Currently, there are no diagnostic criteria for long-COVID, but some symptoms resemble those of fibromyalgia (FM), a chronic disorder characterized by widespread musculoskeletal pain, accompanied with consequent fatigue, cognitive and other somatic symptoms^9,10^. FM is listed by the CDC as one of the conditions sharing symptoms with long-COVID^11^ and recent studies showed that 30–40% of individuals with long-COVID meet the American College of Rheumatology (ACR) FM diagnostic criteria^12,13^.

FM’s underlying cause has not been proven, but it is hypothesized to involve central sensitization, possibly attributed to neurochemical imbalances and dysregulated immune responses triggered by numerous factors, including viral infections^14–16^. Some researchers postulate that Sars-Cov-2 may be associated with persistent neuroinflammation and microglial overactivation^17–19^. This reaction may impair the brain connectivity between pain-processing regions and sensorimotor areas, leading to reduced functional connectivity and alterations of both white and grey matter^20–23^.

The unknown origins and atypical pain patterns associated with FM in long-COVID individuals make its approach challenging^13^. Current therapies, including anti-inflammatory^24^ and central pain-targeted agents have shown limited efficacy^25^, with few drug-based randomized clinical trial (RCTs) demonstrating pain relief in individuals with long-COVID and FM^26^. This highlights the need for effective therapies in this population.

Transcutaneous electrical nerve stimulation (TENS) is a supportive intervention believed to alleviate pain by stimulating peripheral nerves and modulating central pain processing^27,28^, reaching the CNS through a sequenced stimulation of non-painful 1st -order neurons (Alpha-beta fibers) leading to the activation of pain inhibitory interneurons in the dorsal horn (referred as “nerve gate”)^29^. This hypothesized effect blocks pain signals between painful 1st -order neurons (A-delta and C-fibers) and 2nd -order neurons (spinothalamic tract)^30,31^. A recent meta-analysis encasing 11 randomized controlled trials (RCT) involving patients with FM undergoing TENS therapy reported a large mean reduction in pain influenced by higher number of sessions, frequency, and intensity^32^. However, this technology’s effect on pain has not been explored in individuals with long-COVID developing FM-like symptoms.

This pilot study investigated wearable TENS therapy as a potential treatment for FM-like symptoms in individuals with long-COVID. We hypothesize that 4-weeks of daily home-based wearable TENS therapy will reduce pain and fatigue interference with functional outcomes, and gait alterations among this population. In addition, we anticipate this regimen will be feasible and acceptable.

## Methods

### Study population

A pilot RCT of individuals with long-COVID was conducted between April 2022 and August 2023. Participants were referred by a pulmonologist and critical care specialist from the post-COVID-19 Care Clinic or Internal Medicine Clinic at Baylor College of Medicine (Houston, TX), or self-referred by meeting the criteria in our public ClinicalTrials.gov registered protocol (Identifier: NCT05200858, 01/21/2022). All participants signed an informed consent approved by the local Institutional Review Board (IRB number: H-50753) before study enrollment. The study followed the Consolidated Standards of Reporting Trials (CONSORT) guidelines for RCTs. The methods used were in accordance with the relevant guidelines and regulations, and the Helsinki Declaration.

Inclusion criteria were: 18–64 years old; reported persistent musculoskeletal pain, fatigue, and/or weakness in one or more body sections that were not present before acute COVID-19 infection; had access to a personal smartphone and willing to install a smartphone application; and able to attend in-person visits. Exclusion criteria were patients with demand-type cardiac pacemaker; implanted defibrillator; major lower extremity wounds; and previous neuromuscular diseases (i.e., Guillan-Barre, Myasthenia Gravis, multiple sclerosis) or hearing weakness.

Baseline demographics, comorbidities, and current long-COVID symptoms were gathered from participants’ medical records. Neurovascular baseline characteristics, including bilateral sural nerve conduction velocity and amplitude, and plantar tissue oxygenation (SatO2), were assessed using the DPNCheck (NeuroMetrix Inc., MA, US) and SnapShot NIRS (Kent Imaging, Al, CAN) devices, respectively.

### Randomization, group allocation, and intervention

**Fig. 1:**
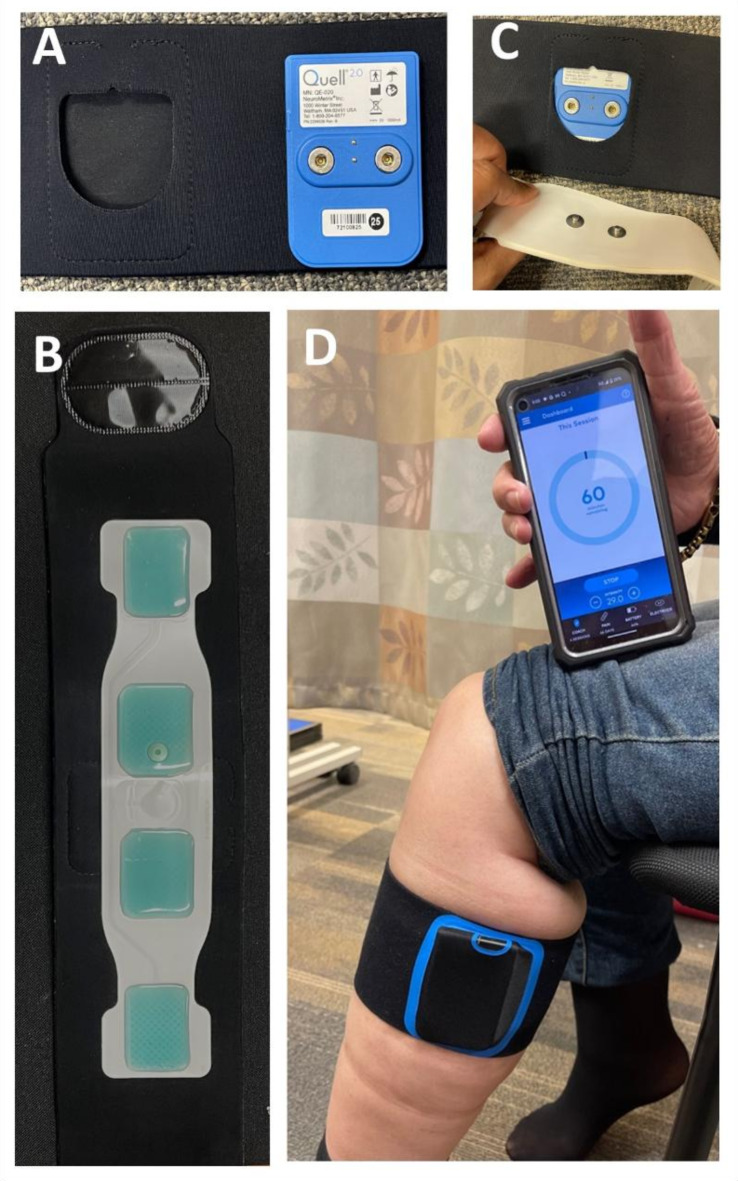
Transcutaneous nerve electrical stimulation device set up and placement. (**A**) TENS device located in the band-strap, (**B**) Hydrogel pad attached inside band-strap, (**C**) Hydrogel pad connected to the device located inside the band-strap, (**D**) Band-strap (containing the device and hydrogel) attached around the upper-calf and paired to a smartphone via Bluetooth.

Participants were randomized (ratio: 1:1) to intervention (IG) and placebo (PG) groups through a computer-generated list followed by sequential allocation. Participants and care providers were blinded to the group allocation. Investigators collecting and analyzing data were not blinded. The IG received high-dose (1-hour) TENS therapy utilizing an FDA-cleared wearable device (Quell, NeuroMetrix Inc., MA, US). The device was unilaterally attached around the patients’ upper calf via four hydrogel pads containing an electrode array, secured to a stretchable band strap (Fig. 1). The device consists of a one-channel electrical stimulator that communicates with a smartphone application through Bluetooth. The PG was provided with an identical device that elicited 10% dose (6-minutes per hour) of TENS therapy.

Participants self-administered therapy daily, each session lasting 1 h. They were instructed to complete 3–5 sessions per day, alternating device placement contralaterally weekly. After the initial 4 weeks, participants were unblinded, with the IG continuing TENS therapy for an additional 4 weeks (total of 8 weeks), while the PG switched to a high-dose TENS device for the subsequent 4 weeks. Weekly support phone calls addressed device-related queries. No lifestyle/dietary modifications to pain medication were enforced during the study.

### Device and smartphone app characteristics

The system around the upper calf provides a total stimulation surface area of 60 cm^2^, stimulating sensory dermatomes S2 through L4, commonly targeted for lower body pain^33^. The stimulator generates bipolar, current-regulated pulses with a duration of 290 microseconds and alternating leading phase polarity. Stimulation frequency uniform distribution between 60 and 100 Hz^34^. These device’s characteristics were proven safe in a recent study of patients with FM^35^. The mobile application serves as a remote control for stimulator functions.

### Procedures and measurements

Participants attended three in-person visits at our facility during regular work hours. Assessments for both groups occurred at baseline, 4 weeks, and 8 weeks. At each visit, pain, fatigue, and gait were evaluated.

### Outcomes

The primary outcome included functional interference from pain, assessed via the Brief Pain Index questionnaire interference composite score (BPI-I)^36^. Secondary outcomes included: (1) pain severity, assessed via the BPI questionnaire severity composite score (BPI-S)^36^; (2) fatigue, via the multi-dimensional assessment of fatigue (MAF) questionnaire, and calculating the Global Fatigue Index (GFI)^37,38^; and (3) gait measured using five inertial measurement units (IMUs, LEGSys BioSensics, MA, USA) on participants’ ankles, thighs, and waist. Parameters included stride time^39^, cadence^40^, and double-support phase^41^, and were measured during three consecutive tasks: (1) simple-task (30-feet walk at normal pace); (2) dual-task (30-feet walk at normal pace while counting aloud backwards 2); and (3) fast-walking-task (30-feet walk at a faster pace without jogging/running)^42^. In case of marked fatigue during the walking tasks, patients completed questionnaires via RedCap at home.

### Feasibility metrics

Compliance to the TENS device was linked to a cloud system. The median therapy sessions per day and the median days of device use were quantified. Completers were those adherent to the TENS device for ≥ 3 sessions per day. High compliance was set to 70%^43^ of the maximum possible number of sessions per day (3.5 out of 5) and days in 4-weeks (21 out of 30). Adverse events and therapy discontinuation were reported.

Acceptability to the TENS device was assessed using a Technology Acceptance Model (TAM) questionnaire^44^ (Supplemental Table 1), that measured: perceived usefulness (PU), perceived ease of use (PEOU), and attitude towards use (ATU).

### Power analysis

G*Power software (version 3.1.6) calculated the minimum sample size based on a similar study^35^. The following parameters were used: (1) effect size d = 1.168^45^, (2) 80% power, (3) 5% alpha, (4) two groups, and (5) equal number of participants per group. A minimum of 13 subjects per group, totaling 26 subjects, is required. A sample size of *n* = 12 per group is expected to yield 95% power for detecting a time-effect difference. To accommodate an anticipated 10% dropout rate, we aimed to enroll 30 subjects (15 per group) in this study.

### Statistical analysis

Categorical data was presented as number and percentage (%). Shapiro–Wilk test determined normality of continuous data (*p* > 0.05). Normally distributed data was presented with mean ± standard deviation; non-normally distributed data with median (interquartile range, IQR). Baseline characteristics between groups were compared using t-test, Chi-square, and Mann-Whitney U test, with Cohen’s d for effect size. Interaction effect between group and time was analyzed using generalized estimation equations (GEE) representing estimated means and standard errors [SE] at baseline and 4-weeks. No covariates adjustments were made due to low sample size. TAM % agreement per item was calculated using our prior medical technology acceptability studies’ Equation^46–48^, then compared between groups using t-test. SPSS 29.0 (IBM) was utilized for all statistical analyses with a significance set at *p* < 0.05.

## Results

**Fig. 2:**
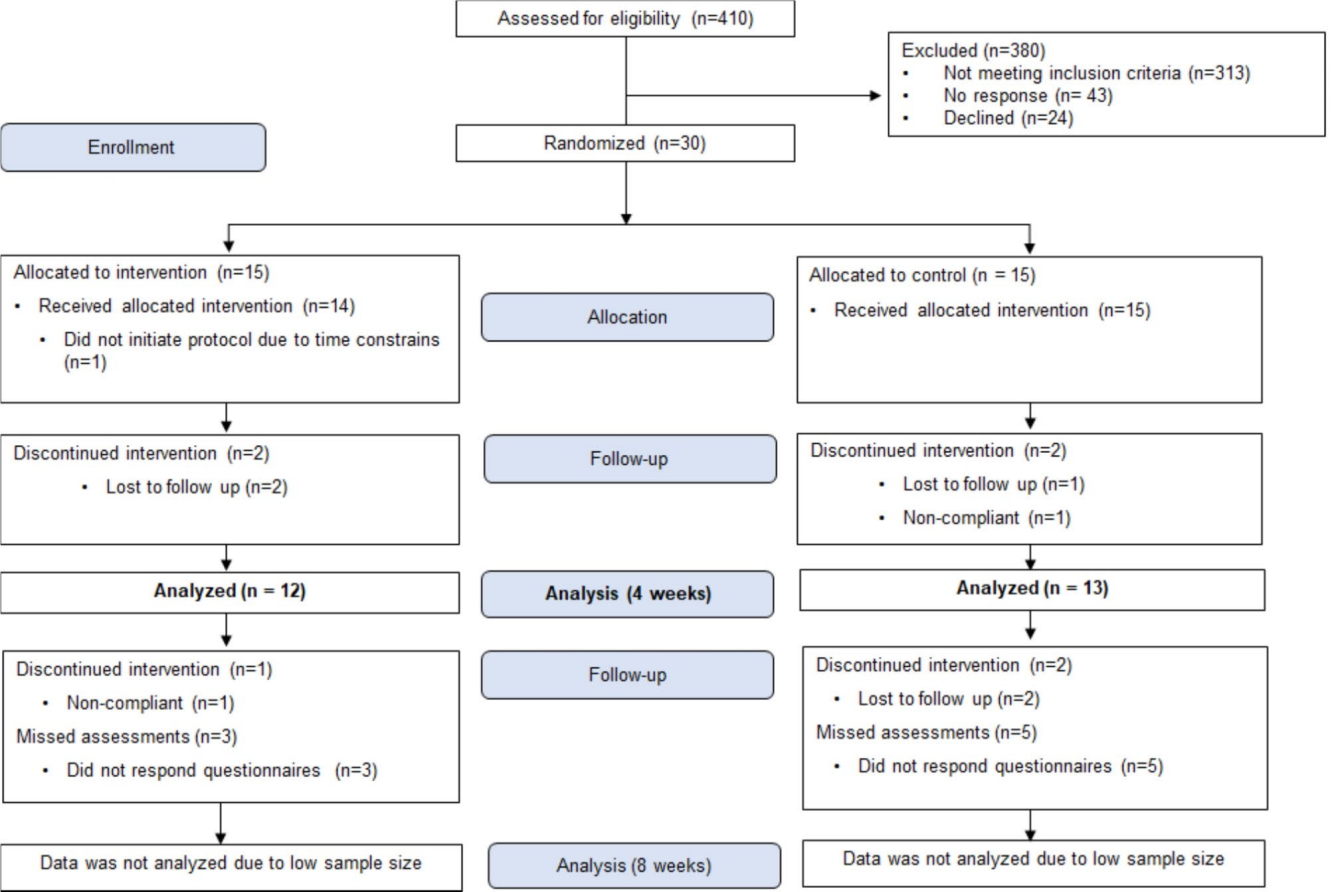
Patient flow chart.

Thirty participants met the criteria, but five discontinued intervention during the blinded study phase (4-weeks), as detailed in Fig. 2. Thus, 25 patients were included for analysis (IG, *n* = 12; PG, *n* = 13). Twenty-three patients were referred from our post-COVID-19 Care/Internal Medicine Clinic; two patients were self-referred. The unblinded phase of the study (week 4 to 8) was not analyzed due to the increase in lost to follow-ups (*n* = 4) and missed final visits (*n* = 7).

### Patient characteristics

Baseline clinical characteristics revealed a higher incidence of cancer history (41.7% vs. 0%, *p* = 0.03) and osteoarthritis (33.3% vs. 0%, *p* = 0.01) in the IG compared to the PG. Other baseline characteristics did not significantly differ between groups (Table 1).

**Table 1:** Baseline clinical characteristics.

	Placebo group (*n* = 13)	Intervention group (*n* = 12)	*P*-value	Effect-size (Cohen’s d)
Demographics				
Age, years	43.4 ± 11.8	51 ± 12.3	0.12	0.64
BMI, kg/m^2^	27.7 ± 7.9	27.8 ± 6	0.98	0.01
Sex, female	9 (69.2)	10 (83.3)	0.41	0.33
Race, no.				
African American	1 (7.7)	2 (16.7)	0.36	1.06
Hispanic	2 (15.4)	3 (25)		
Asian	2 (15.4)	0		
White	6 (46.2)	6 (50)		
Other	2 (15.4)	1 (8.3)		
Comorbidities				
High blood pressure, no.	3 (23.1)	3 (25)	0.91	0.05
Heart disease	3 (23.1)	5 (41.7)	0.32	0.41
Depression	3 (23.1)	3 (25)	0.91	0.05
Osteoarthritis	0	4 (33.3)	0.03	0.98
Cancer	0	5 (41.7)	0.01	1.22
Brain fog	7 (53.9)	9 (75)	0.27	0.45
Hospitalization due to COVID-19	5 (38.5)	4 (33.3)	0.79	0.11
Admitted to ICU	2 (15.4)	0	0.16	0.59
Supplemental oxygen hospital	4 (30.8)	1 (8.3)	0.16	0.58
Current physical therapy	2 (15.4)	3 (25)	0.61	0.21
Neurovascular lower extremity features				
Sural Nerve conduction velocity, m/s	58.1 ± 6.6	55.9 ± 4	0.19	0.39
Sural Nerve amplitude, µV	14.1 ± 9.5	13.9 ± 5.8	0.91	0.02
Plantar SatO2, %	66.6 ± 3.9	67 ± 6.2	0.72	0.08
Current long-COVID symptoms				
Days of persistent symptoms	335.5 ± 175.6	377.7 ± 249.1	0.63	0.28
Memory difficulty, no.	10 (80)	11 (91.7)	0.59	0.43
Shortness of breath	7 (53.9)	5 (41.7)	0.70	0.51
Fatigue	13 (100)	11 (91.7)	0.31	0.43
Insomnia	6 (46.1)	7 (58.3)	0.78	0.43
Weakness	11 (84.6)	9 (75)	0.33	0.64
Muscle pain	11 (84.6)	8 (66.7)	0.42	0.72
Unsteady gait	7 (53.9)	6 (50)	0.76	0.45
Atrophy	8 (61.5)	5 (41.7)	0.27	0.71
Numbness	11 (84.6)	6 (50)	0.07	1.08

Reported as mean ± standard deviation or n (%). Reported symptoms were not present before acute COVID-19 infection.

*Kg* kilograms, *m * meters,* no.* number,* µV* microvolts,* m* meters,* s* seconds, muscle pain refers to pain in one or more muscles at the time of clinical assessment.

### Outcomes

Baseline BPI-I was significantly higher in the PG than the IG (Table 2). At 4-weeks, the IG showed a significantly greater decrease in BPI-I scores compared to the PG (mean difference = 2.61, *p* = 0.008, d = 1.12**)**. However, within-group comparison did not show significant improvement (Table 3). Baseline BPI-S and GFI did not significantly differ between groups (Table 2), nor did they show significant differences at 4 weeks (Table 3).

**Table 2:** Baseline score comparison for pain and fatigue between groups/sub-groups.

	Placebo group (n = 13)	Intervention group (n = 12)	P-value	Effect-size (Cohen’s d)
All cohort				
BPI-I, score	5.54 ± 0.62	3.17 ± 0.81	0.021	3.3
BPI-S	4.79 ± 0.43	3.60 ± 0.69	0.15	2.09
GFI	37.89 ± 3.32	37.75 ± 2.65	0.98	0.05

Fibromyalgia sub-group	Placebo sub-group (n = 9)	Intervention sub-group (n = 5)	P-value	Effect-size (Cohen’s d)
BPI-I, score	6.48 ± 0.65	6.06 ± 0.66	0.65	0.64
BPI-S	5.28 ± 0.47	5.35 ± 1.01	0.95	0.1
GFI	42.63 ± 2.54	43.16 ± 2.38	0.88	0.21

Reported as mean ± standard deviation.

*PI-I* functional interference from pain via brief pain inventory,* BPI-S* pain severity via brief pain inventory,* GFI* global fatigue index via the multi-dimensional assessment of fatigue.

**Table 3:** Pain and fatigue score comparison between groups/sub-groups at 4-weeks.

Variable	N	Mean change	SE	Treatment comparison at 4-weeks (IG vs. PG)		
				Mean difference	95% CI	p-value
All cohort						
BPI-I, score						
PG	12	0.198	0.407	-2.616	-4.543, 0.689	0.008
IG	11	0.454	0.503			
BPI-S						
PG	11	0.289	0.255	-1.305	-2.894, 0.284	0.107
IG	10	1.184	0.815			
GFI						
PG	11	2.231	3.207	-0.732	-9.298, 7.834	0.867
IG	10	2.821	1.504			
Fibromyalgia sub-group						
BPI-I, score						
PG	9	0.079	0.327	-1.397	-3.183, 0.39	0.08
IG	5	1.057	0.603			
BPI-S						
PG	9	0.028	0.164	-0.456	-2.097, 1.186	0.587
IG	5	0.5	0.604			
GFI						
PG	8	1.747	1.503	-3.806	-9.132, 1.52	0.161
IG	5	6.08*	2.188			

*PG* placebo group,* IG* intervention group, *BPI-I* functional interference from pain via brief pain inventory, *BPI-S* pain severity via brief pain inventory,* GFI* global fatigue index via the multi-dimensional assessment of fatigue.

*Significant within group improvement.

Objective assessment of gait parameters showed no significant differences in baseline stride time, cadence, and double support phase between groups (Table 4). At 4-weeks from baseline, there was a significant improvement for stride time during the three walking tasks in the IG (single: 5.83%; dual: 8%; fast-walking: 3.96%, all *p* < 0.05). Similarly, cadence significantly improved at 4-weeks from baseline during the three waking tasks in the IG (single: 5.57%; dual: 9.5%; fast-walking: 3.89%, all *p* < 0.05). A significant time x group effect was seen for cadence and stride time (Supplemental Table 2). In addition, double-support phase significantly improved during the dual-task at 4-weeks from baseline in the IG (11.57%, *p* = 0.017).

**Table 4:** Gait parameters comparison within and between groups at 4-weeks.

Variable	Placebo group (n = 12)+		Time effect p-value	% change	Intervention group (n = 8)*		Time effect p-value	% change	P-value (Cohen’s d) group difference at baseline	P-value (Cohen’s d): group difference at 4-weeks
Single task										
Stride time (sec)	Baseline	1.18 ± 0.04	0.32	-2.54	Baseline	1.20 ± 0.04	0.017	-5.83	0.7 (0.15)	0.56 (0.17)
	4-weeks	1.15 ± 0.04			4-weeks	1.13 ± 0.03				
Double support phase (%)	Baseline	22.82 ± 1.16	0.93	-0.39	Baseline	24.13 ± 0.69	0.179	-4.31	0.33 (0.39)	0.82 (0.1)
	4-weeks	22.73 ± 1.25			4-weeks	23.09 ± 0.93				
Cadence (steps/min)	Baseline	103.22 ± 3.24	0.34	2.10	Baseline	101.40 ± 3.50	0.011	5.57	0.7 (0.17)	0.68 (0.17)
	4-weeks	105.39 ± 3.12			4-weeks	107.05 ± 2.59				
Dual task										
Stride time (sec)	Baseline	1.39 ± 0.12	0.06	-11.51	Baseline	1.25 ± 0.05	0.003	-8.00	0.26 (0.42)	0.33 (0.38)
	4-weeks	1.23 ± 0.07			4-weeks	1.15 ± 0.05				
Double support phase (%)	Baseline	28.02 ± 2.68	0.34	-6.64	Baseline	25.32 ± 1.33	0.017	-11.57	0.37 (0.35)	0.24 (0.45)
	4-weeks	26.16 ± 2.96			4-weeks	22.39 ± 1.27				
Cadence (steps/min)	Baseline	91.68 ± 5.25	0.09	9.95	Baseline	97.27 ± 3.61	0.005	9.50	0.38 (0.36)	0.42 (0.36)
	4-weeks	100.80 ± 4.82			4-weeks	106.51 ± 5.29				
Fast-walking task										
Stride time (sec)	Baseline	1.05 ± 0.04	0.77	-1.90	Baseline	1.01 ± 0.04	0.048	-3.96	0.51 (0.3)	0.26 (0.5)
	4-weeks	1.03 ± 0.04			4-weeks	0.97 ± 0.03				
Faster walking: double support phase (%)	Baseline	19.62 ± 1.77	0.32	-7.85	Baseline	20.29 ± 0.94	0.065	-12.12	0.74 (0.13)	0.9 (0.05)
	4-weeks	18.08 ± 1.54			4-weeks	17.83 ± 1.13				
Faster walking: Cadence (steps/min)	Baseline	116.67 ± 3.94	0.71	1.59	Baseline	120.67 ± 4.43	0.019	3.89	0.5 (0.3)	0.3 (0.45)
	4-weeks	118.53 ± 4.71			4-weeks	125.36 ± 4.64				

Reported as mean ± standard deviation. Sec, seconds; min, minutes.

*Sec* seconds,* min* minutes.

+One patient did not complete the tasks either at baseline or 4-weeks.

*Four patients did not complete the tasks either at baseline or 4-weeks.

### Fibromyalgia sub-group

Participants meeting the 2010 ACR FM diagnostic criteria^49^ were labeled as the FM sub-group (PG-FM, *n* = 9; IG-FM, *n* = 5). At 4-weeks from baseline, the IG-FM showed a decreased trend for BPI-I scores (mean change = 1.057 [SE = 0.6], *p* = 0.080). Comparison between sub-groups at 4-weeks showed a trend in favor of the IG-FM compared to the PG-FM (mean difference = 1.39, *p* = 0.125, d = 2.257, Table 3). Moreover, the IG-FM showed a significant improvement in GFI scores at 4-weeks from baseline (mean change = 6.08 [SE = 10.61], *p* = 0.005).

### Feasibility metrics

At 4-weeks, median TENS therapy sessions per day were 4 (IQR = 3-4.9) in the IG and 3.5 (IQR = 3–5) in the PG. The median days of device use at 4-weeks were 27 (IQR = 25-27.5) in the IG, and 26 (IQR = 20–27) in the PG. Completers (≥ 3 sessions per day) rate in the IG was 100%, while 92.3% in the PG. High compliance (≥ 3.5 sessions per day) rate in the IG was 54.5%, while 61.5% in the PG (Supplemental Table 3).

No severe adverse events requiring discontinuation were reported. However, two patients (PG, *n* = 1; IG, *n* = 1) experienced mild pain and itchiness, respectively, when TENS was delivered immediately after shaving their lower extremities. After using TENS daily for 5 h, one patient (PG, *n* = 1) reported mild fatigue, and one patient (IG, *n* = 1) reported skin irritation. Two patients (IG, *n* = 1; PG, *n* = 1) complained of pain and skin spots, respectively, from the hydrogel pads when not weekly alternating the device to the contralateral calf.

The TAM questionnaire showed overall acceptability above 70% in all categories, with no significant differences between groups: PEOU (IG = 94.2 ± 10.8% vs. PG = 91.5 ± 9.9%, *p* = 0.42), ATU (IG = 89.2 ± 10% vs. PG = 83 ± 14.4%, *p* = 0.283), and PU (IG = 71.2 ± 11.6% vs. PG = 61.4 ± 14.9%, *p* = 0.13).

## Discussion

This pilot RCT examined the effectiveness of daily wearable TENS therapy in alleviating pain, fatigue, and gait alterations in individuals with long-COVID. High-dose TENS participants experienced greater reduction in functional interference due to pain compared to low-dose TENS participants. Additionally, objective assessment of gait parameters revealed significant improvement in stride time and cadence at 4 weeks from baseline in the high-dose TENS group across various walking tasks. Moreover, both groups exhibited high compliance rates (≥ 3 h per day) and reported device acceptability above 70% during the blinded study phase.

Previous RCTs utilizing FDA-approved pharmacological treatment for FM (i.e., duloxetine^50^, milnacipran^51^) have shown improvement in functional interference from pain when compared to placebo, with a BPI-I — reflecting pain’s impact on general activities, work, walk, relationships, mood and enjoyment of life — between-group mean difference ranging from 0.58 to 1.74 in a 12-week timeframe; however, accompanied by notable side effects including nausea (> 36%), constipation (> 14.7%), and dizziness (> 10.5%), amongst others^49,51^. The present study explored TENS as a safer pain management intervention with known minimal side effects^52^. We acknowledge the IG had less severe functional baseline pain scores than the PG, thus, evidence suggests this cohort would have had lesser improvement post-intervention^53^. However, after 4-weeks of TENS therapy, the IG showed significantly greater reduction of BPI-I scores compared to the PG (mean difference = 2.61 points, *p* = 0.008, Table 2).

The Initiative on Methods, Measurement, and Pain Assessment in Clinical Trials (IMMPACT) suggests a 1-point mean change from baseline to the targeted endpoint as a meaningful clinically significant difference (MCID)^54^. In our study, the FM subgroup undergoing high-dose TENS showed a trend for reaching this MCID as early as 4-weeks (mean change = 1.06 points, *p* = 0.080). This magnitude of reduction in BPI-I scores with high-dose TENS mirrors the improvements noted by Kong et al.^55^ and Jamison et al.^35^ in non-long-COVID patients with FM after 60-days and 3-months therapy, respectively. Our study demonstrates the benefit of high-dose TENS for relieving functional interference from pain within a short period in long-COVID patients who met the criteria for FM. Despite a small sample size, the substantial effect size compared to low-dose TENS at 4-weeks (d = 2.26) indicates the potential of high-dose TENS for larger, longer-term studies.

Functional interference from fatigue is also crucial to be assessed in long-COVID patients given its prolonged and severe nature affecting activities of daily living^56^.Therefore, we used the GFI, a reliable tool that widely assess the impact of fatigue in many detailed aspects of life, from simple household duties and running errands, to social factors, recreational activities, exercising, sexual activity, amongst others^37,38,57,58^. In patients with FM, GFI has shown significant improvement (1.4 to 1.6 points) from baseline to 14-weeks in those taking mild-moderate pregabalin doses (300 mg to 450mg)^59^, with further improvement when increasing doses (600 mg) over 32 weeks^60^. Interestingly, other pregabalin trials identified that substantial fatigue reduction, such as 10-point GFI decrease, was only seen in FM patients experiencing a 30% of pain reduction^61^. This trend was echoed in a recent study employing 4-week TENS therapy in women with FM^62^, which demonstrated a significant GFI improvement of 4.6-points correlated with reduced pain evoked by movement. While our study showed a significant 6.08-point GFI improvement in the sub-group of FM patients receiving high-dose TENS at 4-weeks from baseline, this mean change did not correlate with the observed changes in BPI-I scores. We attribute this discrepancy to the insufficient sample size, which limited our ability to distinguish between FM long-COVID patients experiencing major versus minor pain reduction alongside significant GFI improvement. In addition, despite there is some overlap between the GFI and BPI-I outcomes — working, walking, relationships/socialization—the GFI includes more areas like exercising, sexual activities, cooking, bathing, amongst others, that may be more influenced by fatigue than pain. A larger sample size evaluating similar domains is needed to determine the relationship between fatigue and pain improvement in long-COVID patients undergoing high-dose TENS.

In a recent systematic review, significant gait alterations were found in FM patients^63^. Utilizing instrumented walkway systems, observational studies revealed FM patients have significantly shorter cadence and longer double support phases compared to healthy individuals^64^. The present study exhibited that long-COVID patients with widespread pain undergoing 4-week high-dose TENS significantly improved their cadence by 5.6% and 9.5% in the single- and dual-tasks, respectively, and 3.9% in the fast-walking task. Moreover, studies have incorporated cognitive elements (dual-task) in walking assessments to predict risk-of-fall caused by fatigue in FM patients^65^, showing a notable impact on double-support phase during such tasks^66^. In the present study, the double-support phase of long-COVID patients undergoing 4-weeks high-dose TENS significantly improved by 11.6% in the dual-task. While no MCID is established for gait via wearables in FM patients, Kaleth et al.^67^ established an anchor-based MCID for walking distance, indicating clinically meaningful fatigue improvement. Our findings suggest that reducing functional interference from fatigue may aid fall prevention in long-COVID patients with chronic pain; however, further risk-of-fall assessment is needed for confirmation.

To date, the objective assessment of gait improvement in FM patients has been limited to studies focusing on interventions like aerobic exercise and physical therapy^68–70^. Moreover, the effectiveness of TENS in enhancing gait has only been investigated in stroke patients, however incorporating exercise therapy into the treatment regimen therapy as well^71^. This modality is often unsuitable for long-COVID patients given their high-risk for post-exertional malaise^72^. Consequently, they are generally advised to refrain from moderate physical exertion^73^. In this context, high-dose TENS offers a promising alternative for improving gait parameters in long-COVID patients with FM-like symptoms. Nevertheless, further research with a larger sample size is essential to substantiate this potential benefit.

The adherence to analgesic drugs among FM patients can be influenced by intermittent pain and adverse events^74^. In a duloxetine 12-week trial, up to 21% participants discontinued therapy due to adverse events^75^. The present study showed no discontinuations at 4-weeks, despite 6 patients reporting mild fatigue, pain, and skin irritation. These issues were resolved with advice from the research team. Compliance resulted in a median of > 3.5 therapy sessions per day and > 26 days of device usage. High compliant (≥ 21 of days used) patients’ rate was 100% in the IG and 81.8% in the PG. However, compliance decreased in the unblinded study phase (week-4 to 8). We attribute this to disengagement from placebo devices, depression, brain fog, and multiple hospital visits encountered by our patients during the study^76^.

A key factor contributing to high compliance may be high acceptability and perceived benefit of the wearable TENS device. The high-dose TENS group showed slightly higher perceived usefulness compared to the low-dose group (~ 71.2% vs. ~ 61.4%), consistent with a previous RCT in FM patients undergoing TENS therapy for 3 weeks^77^. Moreover, all other acceptability items were similar to our previous trial involving long-COVID individuals undergoing electrical stimulation for musculoskeletal sequelae^78^.

### Limitations

This study encountered limitations, including a small sample size and substantial missing data (n = 11 patients) in the second/unblinded phase (weeks 4 to 8) due to challenges such as patients managing in-person clinic visits and coordinating appointments with specialists in pulmonology, cardiology, and rheumatology. The prevalent ‘brain fog’ hindered participants from remembering study-related tasks, impacting questionnaire completion and appointment tracking. The short TENS therapy duration and unanalyzed adjuvant medications effects for pain and fatigue added complexity. While five patients (PG, *n* = 4; IG, *n* = 1) received concurrent physical therapy, detailed session information was not collected. Unblinding at the 4-week visit may have increased loss to follow-up and decreased therapy adherence.

## Conclusion

High-dose daily TENS therapy in individuals with long-COVID experiencing persistent pain, fatigue, and weakness for an average of ~ 356 days post-acute COVID-19 infection was feasible, and acceptable, with a greater improvement in functional interference from pain when compared to low-dose TENS therapy. Additionally, using high-dose TENS led to an improvement in gait characteristics such as cadence, stride time, and double-support phase as early as 4-weeks. Lastly, those individuals with long-COVID meeting the 2010 ACR FM diagnostic criteria showed improvement in functional interference due to fatigue at 4-weeks from baseline. Future studies, with larger sample sizes and extended follow-up periods, are needed to confirm these findings.

## Electronic supplementary material

Below is the link to the electronic supplementary material.

Supplementary Material 1

## Data Availability

The data that support the findings of this study are not publicly available but are available from the corresponding author BN, najafi.bijan@gmail.com upon reasonable request.
