# Supplementary material for: Transcutaneous electrical nerve stimulation for fibromyalgia-like syndrome in patients with Long-COVID: a pilot randomized clinical trial
Source: Sci Rep. 2024 Nov 8;14:27224. doi: 10.1038/s41598-024-78651-5 (PMC11549448; doi:10.1038/s41598-024-78651-5)
Supplement: Supplementary file 1 — Supplementary Material 1 [file 41598_2024_78651_MOESM1_ESM.docx]

**Supplemental Table 1****.** Technology Acceptance Model questionnaire evaluated at 4 weeks,

before unblinding participants.

| **Perceived ease of use (PEOU)** | **Please rate your experience with the following:** | **Very difficult (1)** | **Difficult**  **(2)** | **Neutral**  **(3)** | **Easy**  **(4)** | **Very Easy**  **(5)** |
| --- | --- | --- | --- | --- | --- | --- |
|  | Device was easy to use |  |  |  |  |  |
|  | Hydrogel pad was easy to use |  |  |  |  |  |
| **Perceived usefulness (PU)** | **Please indicate the influence of the device on:** | **Increased significantly (1)** | **Increased**  **(2)** | **Neutral**  **(3)** | **Decreased (4)** | **Decreased significantly (5)** |
|  | Device decreased pain |  |  |  |  |  |
|  | Device decreased fatigue |  |  |  |  |  |
|  | Device decreased weakness |  |  |  |  |  |
| **Attitude towards use (ATU)** | **Please indicate if you agree or disagree with the following statements:** | **Strongly disagree**  **(1)** | **Disagree**  **(2)** | **Neutral**  **(3)** | **Agree**  **(5)** | **Strongly Agree**  **(5)** |
|  | I would continue this therapy after completing the study |  |  |  |  |  |
|  | I would recommend this therapy to friends and family. |  |  |  |  |  |

**Supplemental Table 2.** Main and interaction effects for gait parameters at 4 weeks

|  | **Group effect (PG vs IG)** | | **Time effect (BL vs 4W)** | | **Group*time interaction effect** | |
| --- | --- | --- | --- | --- | --- | --- |
| **Variable** | **Cohen's d** | **P-value** | **Cohen's d** | **P-value** | **Cohen's d** | **P-value** |
| **Single task** | | | | | | |
| Stride time (sec) | 0.000 | 0.992 | 1.311 | 0.014 | 0.528 | 0.254 |
| Double support phase (%) | 0.287 | 0.525 | 0.414 | 0.364 | 0.353 | 0.437 |
| Cadence (steps/min) | 0.000 | 0.984 | 1.318 | 0.014 | 0.505 | 0.274 |
| Dual task | | | | | | |
| Stride time (sec) | 0.529 | 0.253 | 1.595 | 0.005 | 0.317 | 0.484 |
| Double support phase (%) | 0.516 | 0.264 | 1.058 | 0.037 | 0.209 | 0.64. |
| Cadence (steps/min) | 0.430 | 0.348 | 1.748 | 0.003 | 0.000 | 0.985 |
| **Fast-walking task** | | | | | | |
| Stride time (sec) | 0.466 | 0.311 | 0.476 | 0.301 | 0.224 | 0.619 |
| Double support phase (%) | 0.057 | 0.899 | 0.971 | 0.051 | 0.200 | 0.656 |
| Cadence (steps/min) | 0.438 | 0.339 | 0.558 | 0.229 | 0.235 | 0.602 |

Generalized estimated equation (GEE) was performed to evaluate group*time interaction effect. Sec, seconds; min, minutes.

**Supplemental Table 3.** Compliance data at 4 weeks, before unblinding participants.

|  | **Blinded phase: 4 weeks** | | | | |
| --- | --- | --- | --- | --- | --- |
|  | **Overall compliance** | | **Proportion of adherence** | | |
|  | **(Median)** | | **Completers  N (%)** | **High compliance >70%  N (%)** | |
|  | Sessions per day | Days used | ≥3 Sessions per day | ≥3.5 sessions per day | ≥21 days used |
| **PG**  **(n=13)** | 3.5 [3-5] | 26 [20-27] | 12 (92.3%) | 8 (61.5%) | 9 (81.8%) |
| **IG**  **(n=11)*** | 4 [3-4.9] | 27 [25-27.5] | 11 (100%) | 6 (54.5%) | 11 (100%) |

*Missed data of 1 patient. Median [IQR], n (%), sessions = hours
